# Advancing collaborative research for health: why does collaboration matter?

**DOI:** 10.1136/bmjgh-2024-014971

**Published:** 2024-09-16

**Authors:** Carla Saenz, Timothy M Krahn, Maxwell J Smith, Michelle M Haby, Sarah Carracedo, Ludovic Reveiz

**Affiliations:** 1 Pan American Health Organization, Washington, District of Columbia, USA; 2 Dalhousie University, Halifax, Nova Scotia, Canada; 3 Faculty of Health Sciences, University of Western Ontario, London, Ontario, Canada

**Keywords:** Global Health, Other study design

## Abstract

The calls for health research to be collaborative are ubiquitous—even as part of a recent World Health Assembly resolution on clinical trials—yet the arguments in support of collaborative research have been taken for granted and are absent in the literature. This article provides three arguments to justify why health research ought to be collaborative and discusses trade-offs to be considered among the ethical values guiding each argument.

**SUMMARY BOX**

- Health research ought to be collaborative in emergency and non-emergency situations.
- Arguments for collaborations in health research are grounded in the values of efficiency, benefit maximisation and equity.
- Health research collaboration can encompass many differences and take place in very diverse settings. The values of efficiency, benefit maximisation and equity do not dictate a formula for research collaborations in specific circumstances.
- It may be necessary to consider trade-offs between these values. One may be justified to depart from (more robust) collaboration in specific circumstances. However, it is never acceptable to compromise respect and fairness to advance research collaboration.

The COVID-19 pandemic has made it clear that the impact of research is not limited to advancing people’s health in the future. Research can also be impactful for current health threats.^1^ Research that was conducted very quickly led to the timely development of effective COVID-19 diagnostic tests, vaccines, therapeutics and public health interventions.^2^ Yet this success story should not obscure challenges in the conduct of COVID-19 research.^3^ For example, multiple repetitive, small trials have consumed an important share of research resources while not being able to yield much-needed knowledge about the efficacy of the interventions under study.^4^,^7^ These challenges have been acknowledged to the extent that there have been various calls for increased collaboration in research,^38^,^10^ along with a World Health Assembly resolution calling for increased coordination of clinical trials.^11^ Furthermore, as part of the response to the mpox emergency, WHO urged for ‘collaborative research’.^12^

The call for research to be collaborative has been ubiquitous, even before the pandemic,^13^,^17^ yet the justification to proceed collaboratively when conducting research is not obvious. What do collaborations add to research? Why should we advance research collaborations, instead of just ensuring that research needs are met? Why are research collaborations described as an ethical imperative, particularly in the context of health emergencies?

In this article, we explain why research collaborations are ethically valuable, provide three arguments to justify why health research ought to be collaborative and discuss trade-offs to be considered among the ethical values guiding each argument.

## An ethical framework for collaborative health research

Health is recognised as a common good^18 19^ that critically affects our life prospects and welfare, even our very survival. Health research is vital for advancing health; it is through the conduct of research that we find cures for diseases and ways to prevent and alleviate suffering. To the extent that we ought to promote health, we ought to promote health research.^20^ The ethical value of health confers ethical value to health research.

Therefore, health research is not an ethically neutral activity, that is, one that is impartial to what is ethically valuable and as such optional. Research is an ethically loaded undertaking because it is crucial to advance our common good.^21^ While the connection between research and our health and well-being has been globally palpable during the pandemic, the impact of health research on our health and well-being transcends emergency circumstances because our health and well-being are also threatened by numerous other diseases and health conditions.

This understanding of health research as an ethical endeavour frames the discussion about collaborations in health research. Specifically, the ethical character of health research dictates the ways in which it should be conducted. Whether we conduct this research faster or slower, with greater impact or lesser impact, reaching everybody or only a few with its benefits is not ethically neutral, because something as important as our health and well-being is at stake. That is, since conducting health research constitutes an ethical duty, the way in which that duty is discharged is ethically relevant; it can be done in a way that is more right or wrong, more good or bad.^22 23^

## Arguments for collaboration in health research

Health research is a complex enterprise that is conducted in vastly diverse settings. Accordingly, in emergency and non-emergency situations, it can encompass collaborations among very different parties, including governmental entities, academia, pharmaceutical companies, international organisations and non-governmental organizations (NGOs). Collaborating institutions may be in the same jurisdiction or in distant countries. They may also be very differently resourced, even when they are located within the same country.

The collaborative conduct of health research can entail the following: (1) seeking the involvement of researchers in the locations where research is going to be conducted, (2) seeking the involvement of researchers in the locations where the research results are expected to be beneficial, (3)seeking the involvement of researchers conducting similar studies to avoid duplication and (4) seeking the involvement of researchers with relevant expertise, whether or not they are in the locations where research is going to be conducted or where the research results are expected to be beneficial.^24 25^

Ideally, collaborative research should entail all those four types of collaboration, and more robust collaboration, understood as collaboration that entails more of these types, is in general preferable over less collaborative research. A common path for health research involves a lengthy process that starts in a laboratory, evolves to trials with human participants, which ultimately prove if an intervention is safe and efficacious and moves forward to studies in real-life scenarios to learn about its effectiveness or the challenges posed by its implementation. The further researchers are in the process of putting into practice research results, the stronger the argument for proceeding collaboratively and pursuing all types of collaboration. However, it may be justifiable for researchers to depart from the ideal scenario that includes the four types of collaborations outlined above, provided there are good reasons to do so.

Nonetheless, it is not obvious why collaborations in health research are not just a matter of researcher preference, convenience or standard practice but an ethical imperative. That health research is ethically valuable does not explain why it *ought* to be conducted collaboratively. One may think that it is only justified to team up with those with the highest knowledge and expertise on the research topic, which tend to be in HICs or high-income settings, thus restricting collaborations to those in geographical proximity or those with certain status and reputation.^26^ While the Nuffield Council on Bioethics’ Research in Global Health Emergencies report has made a key contribution stressing that research collaboration is inherently ethical in emergency situations, specific ethical arguments that apply to emergency and non-emergency settings, along with clarification about the implications of these arguments in particular circumstances are still needed.^17^

There are at least three important reasons why collaboration in health research is ethically valuable and ought to be advanced (see table 1). First, collaborations can help research to be conducted faster. Aggregating data from different research sites can yield statistically meaningful results faster. The urge for speed attracts support in emergencies, although there is no prima facie reason to justify delaying the production of research results for non-emergency situations, for example, to treat cancer or chronic conditions. Indeed, speed in non-emergency times is also important to speed up the attainment of the benefit. Furthermore, teaming up with local researchers may be necessary to be able to conduct studies (eg, to have access to the affected patient population or existing samples) or to be able to navigate ethical and regulatory requirements to initiate and adequately oversee them within reasonable time frames. Collaboration can help reduce unnecessary duplication of efforts.^7^ We refer to these reasons as the efficiency argument for collaboration in research.^927^,^29^

**Table 1:** Reasons for collaboration in health research

Value	Rationale	Main type of collaboration
Efficiency	To expedite the conduct of research	Involvement of researchers in the locations where research is going to be conducted. Involvement of researchers conducting similar studies. Involvement of researchers with relevant expertise.
Benefit maximisation	To facilitate the implementation of research results	Involvement of researchers in the locations where the research results are expected to be beneficial. Involvement of researchers conducting similar studies Involvement of researchers with relevant expertise.
Equity	To build research capacity	Involvement of researchers in the locations where research is going to be conducted. Involvement of researchers in the locations where the research results are expected to be beneficial. Involvement of researchers with relevant expertise.

Second, collaborations can facilitate the implementation of research results. Health research seeks to improve the health and well-being of populations, which are the potential benefits of research, that is, its social value. Yet, this value is realised only when research results are implemented. Teaming up with researchers based in the area where research results are meant to be beneficial establishes relationships and builds trust that can expedite the implementation of research results. Moreover, working closely with local researchers helps in the adjustment of research protocols so they are responsive to the specific needs and priorities of affected communities. This enhances the social value of the study, thus facilitating the uptake of its results. This is the benefit maximisation argument for collaboration in research.

Third, collaborations can help to build research capacity.^25^ There is significantly more research capacity in high-income countries (HICs) than in low- and middle-income countries (LMICs),^30^ where many studies are being conducted (often because they host populations with the conditions being researched) or where research results are meant to be beneficial. This situation is often reproduced within countries because there tends to be a research capacity gap between high-income and low-income settings. Teaming up with researchers from LMICs and low-income settings can enhance their research capacity, which in turn can further the prospects for equity in health. Local research capacity is necessary to lead research that addresses the specific health needs of LMICs and low-income settings, and thus effectively address issues that cause health inequities.^24 31^ This is the equity argument for advancing collaborative research.

## Acceptable and unacceptable trade-offs

Concerns for efficiency, benefit maximisation and equity do not dictate a formula for health research collaborations, that is, for optimally dividing roles and responsibilities among collaborating parties. Research collaboration encompasses a wide set of arrangements ranging from close partnerships to informal cooperative interactions.^32^ In general, the stronger the collaborative ties, the better the collaboration may realise the values of efficiency, benefit maximisation and equity that justify and guide research collaborations. However, these values are realised in specific circumstances that may involve various challenges. Moreover, the values are realised in different time frames, for example, equity may only be realised after a long period of collaboration.

Therefore, these values may need to be balanced against each other in particular circumstances to find the optimal approach to a specific collaboration. This is in general how ethical values direct action in every facet of life: instead of dictating a univocal recipe for action, they guide an analysis that considers specific circumstances. Furthermore, research collaborations are not the only way to realise these values of efficiency, benefit maximisation and equity in global health. Similarly, there may be practical obstacles to realise these values through research collaborations that must be taken into account when assessing the best course of action, for example, administrative hurdles, language barriers or even lack of access to knowledge about local expertise.^33^

In situations like outbreaks or health emergencies that are characterised by the absence of effective countermeasures, it may be justified to give higher priority to efficiency and benefit maximisation, which in turn may call for a less robust collaboration with researchers in affected areas if no prior collaboration has been established. While discussions about research plans and methodology are key components of collaborative research and crucial to build capacity and advance equity, they take time that in these circumstances may be better used expediting the initiation of the study. However, a higher priority should not be confused with absolute priority to any value, for example, a higher priority to efficiency and benefit maximisation in specific circumstances should not preclude equity.

Trade-offs between the values of efficiency, benefit maximisation and equity may be acceptable when discharging the duty to conduct health research collaboratively.^23 34^ Yet it is often the case that the achievement of any one of them is dependent on the others. For instance, benefit maximisation can be threatened if research is not efficient. Equity can be threatened when there is inefficiency (ie, efficiency leads to greater benefits and thus greater equity). Benefit maximisation can be threatened if research is inequitable.

Other trade-offs are, however, ethically unacceptable. Research collaboration must always adhere to the bedrock principles of respect and fairness, which are embedded in the standards of research integrity.^17 35^ For example, it would be unethical to exploit collaborators or fail to give credit when credit is due in order to advance efficiency in the publication of research results. Respect and fairness must never be compromised to advance efficiency, benefit maximisation or equity in research collaborations.

## The way forward

Research is a powerful tool to advance people’s health. Collaboration with researchers from affected areas or areas expected to implement research results is not optional but ethically required to advance the values of efficiency, benefit maximisation and equity. Clarifying the reasons why health research ought to be conducted collaboratively and the values that guide research collaborations is, however, only a first step to foster collaboration in health research. Additional practical guidance is necessary to identify research collaborators and establish effective collaborations that advance the best balance of the ethical values at stake.

As with other aspects of research, no algorithm but actual ethics analysis is needed to elucidate the best course of action, for example, when and why departures from more robust collaborations and trade-offs between the values are justified. Robust collaborative research that realises the values of efficiency, benefit maximisation and equity should be advanced as the moral default, in the way we currently advance research that includes populations historically excluded from research as the default and exclude groups only if justified. Proceeding collaboratively when conducting health research is an ethical duty, even if one may be justified in specific circumstances to depart from it or to adopt limited collaborative arrangements.

In order to advance research collaboration across the globe, it is critical for researchers to know what research is underway and who has relevant expertise and capacities. Transparency in research, which includes registration that feeds into WHO’s International Clinical Trials Registry Platform^36^ and publication of research results in indexed journals, is, therefore, essential. But it does not suffice. Strategies to promote fair, respectful health research collaborations must be developed as part of our global health agenda. While frameworks and models to advance collaborations in health research have been proposed (eg, the Bergen Model of Collaborative Functioning^37^), they have not specified why collaborations in health research are not just an option but *ought* to be pursued. As we move forward, the reasons why health research ought to be collaborative, as shown in this article, should meaningfully guide the strategies to advance such collaborations and address practical challenges of implementing research collaborations, for example, overcoming the various barriers that hamper collaborations.

## Data Availability

No data are available.
